# Triboelectric Rotary Motion Sensor for Industrial-Grade Speed and Angle Monitoring

**DOI:** 10.3390/s21051713

**Published:** 2021-03-02

**Authors:** Xiaosong Zhang, Qi Gao, Qiang Gao, Xin Yu, Tinghai Cheng, Zhong Lin Wang

**Affiliations:** 1 Beijing Institute of Nanoenergy and Nanosystems, Chinese Academy of Sciences, Beijing 101400, China; 2201901027@stu.ccut.edu.cn (X.Z.); gaoqi@binn.cas.cn (Q.G.); 2201801004@stu.ccut.edu.cn (Q.G.); yuxinnick@ccut.edu.cn (X.Y.); zhong.wang@mse.gatech.edu (Z.L.W.); 2 School of Mechatronic Engineering, Changchun University of Technology, Changchun 130012, China; 3 School of Electrical and Electronic Engineering, Changchun University of Technology, Changchun 130012, China; 4 CUSPEA Institute of Technology, Wenzhou 325024, China; 5 School of Materials Science and Engineering, Georgia Institute of Technology, Atlanta, GA 30332-0245, USA

**Keywords:** triboelectric sensors, rotary motion, hybrid electrodes, integrated monitoring system, industrial application

## Abstract

Mechanical motion sensing and monitoring is an important component in the field of industrial automation. Rotary motion is one of the most basic forms of mechanical motion, so it is of great significance for the development of the entire industry to realize rotary motion state monitoring. In this paper, a triboelectric rotary motion sensor (TRMS) with variable amplitude differential hybrid electrodes is proposed, and an integrated monitoring system (IMS) is designed to realize real-time monitoring of industrial-grade rotary motion state. First, the operating principle and monitoring characteristics are studied. The experiment results indicate that the TRMS can achieve rotation speed measurement in the range of 10–1000 rpm with good linearity, and the error rate of rotation speed is less than 0.8%. Besides, the TRMS has an angle monitoring range of 360° and its resolution is 1.5° in bidirectional rotation. Finally, the applications of the designed TRMS and IMS prove the feasibility of self-powered rotary motion monitoring. This work further promotes the development of triboelectric sensors (TESs) in industrial application.

## 1. Introduction

Sensor technology is one of three central pillars in modern information technology [1,2] and is applied widely in the fields of industrial automation, automotive electronics, and communication technology [3,4,5]. It has become an indispensable criterion for measuring the development of scientific research and industrial manufacturing. Among all kinds of mechanical motions, rotary motion is one of the most basic forms in the field of industrial automation, which has always been the research hotspot in the fields of science and engineering applications [6,7,8]. The traditional sensing technologies for rotary motion mainly include optoelectronic transformation [9,10], electromagnetic induction [11], and electrical effects [12]. Moreover, with the continuous improvement of the level of industrial automation and manufacturing requirements, passive sensor technology with self-powered function has attracted more and more attention from researchers all over the world [13,14,15].

The triboelectric nanogenerator (TENG, also called the Wang generator [16]), first proposed by Wang Group in 2012, provided a new approach of converting mechanical energy into electricity [17,18,19]. The output signal of TENGs can well reflect the change of mechanical excitation, and TENG has good adaptability to various forms of mechanical motion, thus it is considered to be the potential solution of self-powered sensing [20,21,22,23,24]. In recent years, researchers have realized a preliminary exploration of rotation sensing and monitoring [25,26,27,28,29]. For instance, Xie et al. integrated a triboelectric sensor into a bearing to achieve rotational speed monitoring of shaft components and applied the sensor to the industrial application [30]. Wang et al. proposed a highly sensitive triboelectric self-powered angle sensor used in the fields of robotic arms and personalized medical care, which has the advantages of high resolution, lightweight, and thin thickness [31]. Previous studies are valuable for the development of triboelectric sensors (TESs) [32,33,34,35]. Furthermore, to achieve a comprehensive description of the basic state of the rotary motion in the industrial field, it is essential to monitor multiple rotation parameters simultaneously by TESs. On this basis, a systematic and integrated assembly can further promote the practical application of TESs in industrial application.

Here, we propose a triboelectric rotary motion sensor (TRMS) with variable amplitude differential hybrid electrodes. The hybrid electrodes comprise a variable amplitude electrode and a differential electrode. The rotation speed and direction can be analyzed through the change of amplitude and period of the A-phase electrical signals generated by the variable amplitude electrode. Moreover, the B-phase sine electrical signals generated by the differential electrode can further improve the sensor’s resolution. To verify the feasibility of the TRMS, a series of experiments are carried out to evaluate the monitoring characteristics of the sensor. In the speed range of 10–1000 rpm and the angle range of 360°, the TRMS can meet the basic requirements of the sensor signal monitoring, and has an angle monitoring with a resolution of 1.5° in both clockwise (CW) and counterclockwise (CCW) rotation. Compared with commercial encoders, the TRMS can achieve good linearity and a low error rate. Based on the industrial applications of the designed integrated monitoring system (IMS), this work can realize real-time monitoring of industrial-grade rotary motion state, and be considered as possessing good prospects for industrial application.

## 2. Materials and Methods

**Figure 1:**
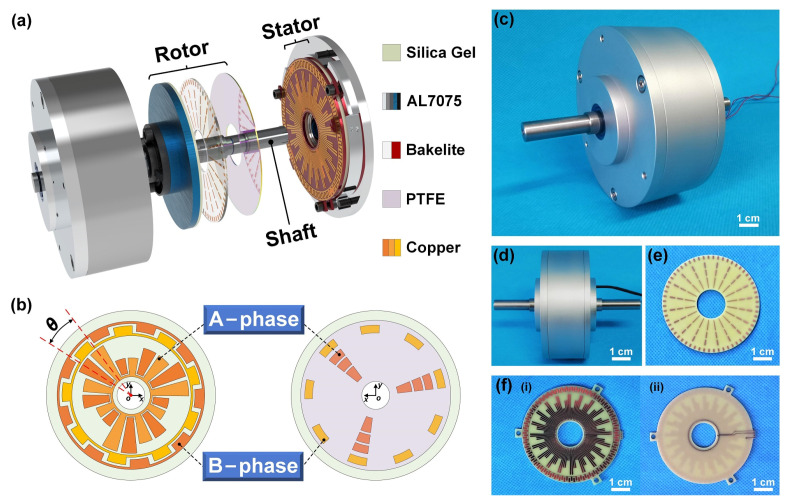
Structural design of the triboelectric rotary motion sensor (TRMS). (**a**) Schematic structure of the TRMS. (**b**) Relative position placement of the electrodes (*θ* = 20°) in the stator and rotor. (**c**,**d**) Photograph of the assembled TRMS. (**e**) Photograph of the printed circuit board (PCB) of the rotor. (**f**) Photographs of front and back of the PCB (*θ* = 3°) of the stator (scale bar: 1 cm). PTFE, polytetrafluoroethylene.

The basic structure of the TRMS is illustrated schematically in Figure 1a, and the prototype is mainly composed of a rotor, a stator, a shaft, and a shell. The rotor consists of a layer of polytetrafluoroethylene (PTFE) film, a printed circuit board (PCB), a silica gel gasket, a turntable, and an adjustment mechanism made of aluminum alloy (AL7075). The stator consists of a silica gel gasket and a PCB. The PCB comprises a layer of copper electrodes with different shapes (thickness 35 μm) and a bakelite disk (thickness 1 mm) through established PCB production technology. A layer of PTFE with a thickness of 80 μm is attached to the surface of the copper electrodes of the rotor. To improve the space utilization and output power of the sensor, each group of variable amplitude electrodes of the stator corresponds to one electrode of the rotor in the A-phase, and each pair of differential electrodes in the stator corresponds to one electrode of the rotor in the B-phase. To better transmit torque, the turntable is used to connect the shaft and the PCB. Besides, the adjustment mechanism of the rotor and the silica-gel gasket can allow the rotor and the stator to more fully come into contact. The rotor and the stator consist of a TENG module, which converts external rotary mechanical energy into electricity based on the coupling of the triboelectric effect and electrostatic induction.

As shown in Figure 1b, the electrodes of the TENG module are divided into two phases: A-phase and B-phase. For the convenience of observation, the interdigital electrode interval *θ* is set as 20°. The stator copper electrodes of A-phase and B-phase are two groups of annular-arranged interdigital electrodes. In the A-phase, every two electrodes of the stator with the same size form a pair of interdigital electrodes. However, the length of each pair of adjacent interdigital electrodes is different. As the absolute value of the output signal is proportional to the friction area, the amplitude of adjacent periodical signals output by the A-phase is variable. When the rotation direction is recognized by the variable amplitude electrical signals, the sensor needs to output at least three period electrical signals with different amplitude. Therefore, every three pairs of interdigital electrodes of the A-phase are set as a group of circulating electrodes. Besides, the B-phase electrodes of the stator are a group of differential interdigital electrodes with the same electrode interval as the A-phase. Further, the signal phases corresponding to A-phase and B-phase are shifted by *θ*/2 along the rotation direction, which can double the sensor’s resolution. When the number of differential electrode groups increases, the sensor’s resolution can be further elevated. It is worth noting that the A-phase electrodes of the rotor are evenly divided into three copper electrodes according to the change in the length of a set of stator variable amplitude electrodes, which can effectively reduce the interference to the amplitude change of the electrical signal.

Figure 1c,d show photographs of the assembled TRMS. The interdigital electrode interval *θ* is processed to 3° under the premise of manufacturing accuracy. Therefore, the resolution of the prototype can reach 1.5°. The PCBs of the rotor and stator are shown in Figure 1e,f, respectively.

**Figure 2:**
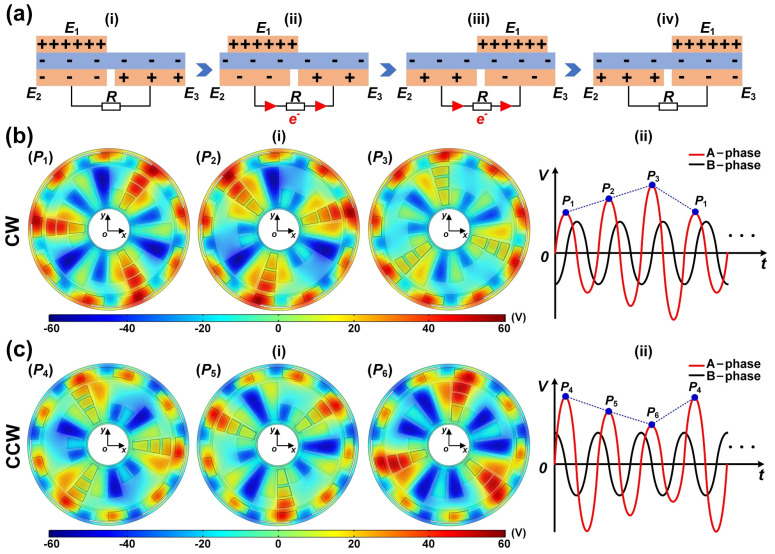
Operating principle of the TRMS. (**a**) The working principle of generating the electrical signals. (**b**,**c**) The finite element simulation of the potential distributions of the TRMS and the sketches of signal generation processes under the different rotation directions. CW, clockwise; CCW, counterclockwise.

The mechanical energy in the TRMS comes from the synchronous rotary motion of the rotor, so the external rotary motion can be monitored by the electrical signal generated by the TRMS. The rotor is in sliding contact with the stator to create electrical signals with two phases simultaneously. Taking the B-phase as an example, the operating principle of generating the electrical signals is shown in Figure 2a.

When the rotor slides by external rotary, the copper electrodes (*E*_1_, *E*_2_, and *E*_3_) in the rotor and stator will generate positive charges under the action of the triboelectric effect. Owing to the different triboelectric polarities, the PTFE film will generate negative charges. The electrode of the rotor *E*_1_ is wholly aligned with the electrode of the stator *E*_2_ (state i). As the PTFE film is pasted on the electrodes of the rotor, the positive charges of the electrode *E*_1_ are equal to the sum of the negative charges on the surface of the PTFE and the electrode *E*_2_. There is no charge transfer between the interdigital electrodes in the stator due to the electrostatic equilibrium. When the electrode *E*_1_ slides from the corresponding position of electrode *E*_2_ to electrode *E*_3_ (state ii to state iii), the original electrostatic equilibrium will be destroyed. Under the action of electrostatic induction, an electric potential difference will be generated between the interdigital electrodes in the stator, which will cause electrons to flow between the interdigital electrodes of the stator to form a new electrostatic equilibrium, and causing the external load to form a transient current. Once the electrode *E*_1_ completely overlaps the electrode *E*_3_ (state iv), all the electrons are transferred to the electrode *E*_3_, and the electrostatic equilibrium between the interdigital electrodes of the stator is reached again. This is the half cycle of the electrical signal generation process. Similarly, when the rotor continues to slide, the electrons will flow back from electrode *E*_3_ to electrode *E*_2_. Therefore, an alternating current signal is generated during the continuous relative sliding of the slider.

Figure 2b(i),c(i) illustrate a cycle rotary process of the TRMS in two different rotation directions and finite element simulation results of the potential distributions using COMSOL software. The corresponding electrical signals are shown in Figure 2b(ii),c(ii).

## 3. Results and Discussion

### 3.1. Output Characteristics

**Figure 3:**
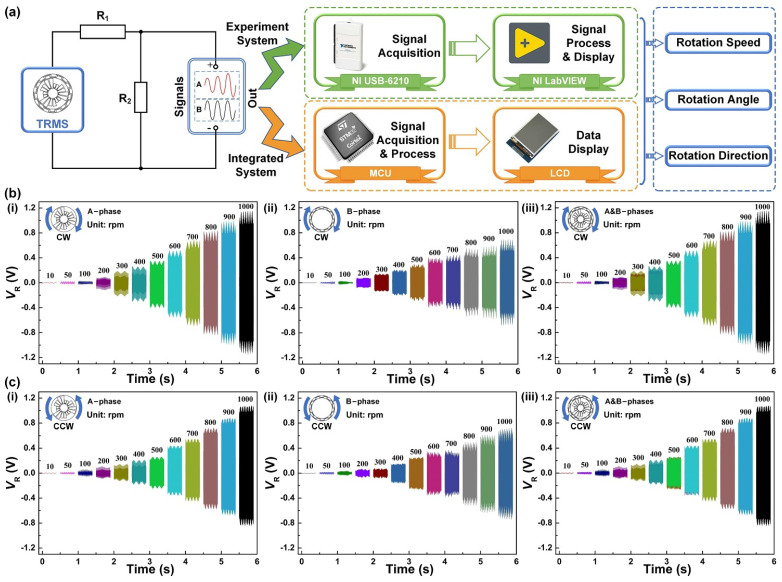
Output characteristics of the TRMS under different rotation speeds. (**a**) The schematics of the experiment system and integrated monitoring system (IMS) of the TRMS. (**b**) The CW rotation: A-phase, B-phase, and A&B-phases. (**c**) The CCW rotation: A-phase, B-phase, and A&B-phases. LCD, liquid crystal display; MCU, microcontroller unit.

In the electrical measurement process of the actual application, the TRMS is driven by a commercial motor at specified rotation speeds. To meet the requirements for the signal processed by the microcontroller unit (MCU) and ensure the authenticity of the analyzed signal, a voltage divider circuit is connected to the TRMS. Among them, the resistance relationship of the voltage divider circuit is R_1_ > R_2_. Resistances of the loads R_1_ and R_2_ of the voltage divider circuit are selected as 200 MΩ and 0.1 MΩ, respectively. To carry out the experiment and application of the TRMS, the electrical signals after passing through the voltage divider circuit can be acquired, processed, and analyzed by two different systems. Figure 3a illustrates the schematics of the experiment system and IMS of the TRMS, respectively. The experiment system acquires electrical signals through a data acquisition card (NI USB-6210) and uses NI LabVIEW software to process the electrical signals. The relevant information such as the rotation speed, angle, and direction of external rotary motion is obtained by analyzing the electrical signals and finally displaying them on the software interfaces. To realize the integrated application of rotary motion monitoring in actual working states, the IMS uses an MCU for electrical signal acquisition, processing, and analysis. A liquid crystal display (LCD) is used to visually display the rotation speed, angle, and direction of the rotary motion processed by the MCU.

To verify the basic output performance of the TRMS, the experiment system is used to conduct experimental tests in the CW and CCW rotation. Figure 3b,c show the output performance of the variable amplitude electrode (A-phase (i)) and the differential electrode (B-phase (ii)) of the TRMS in the speed range of 10–1000 rpm, respectively. It can be seen that the load voltage under the different rotation speeds meets the signal processing requirements of the MCU, which can avoid saturation distortion during signal processing and reduce signal analysis errors. Meanwhile, the load voltage of the electrical signal increases with the raising of the rotation speed. As rotation speed monitoring utilizes the number of electrical signal periods as a reference, the change of load voltage amplitude does not affect the signal processing process. Moreover, Figure 3b(iii),c(iii) show the hybrid electrical signals generated by the two phases of TRMS. Because the output electrical signals are acquired through two channels that do not interfere with each other, the hybrid electrical signals output by the hybrid electrodes is steadily leading to no effect on signal processing and analysis. According to the experimental results, the TRMS can achieve effective capture of rotary mechanical energy in the speed range of 10–1000 rpm.

**Figure 4:**
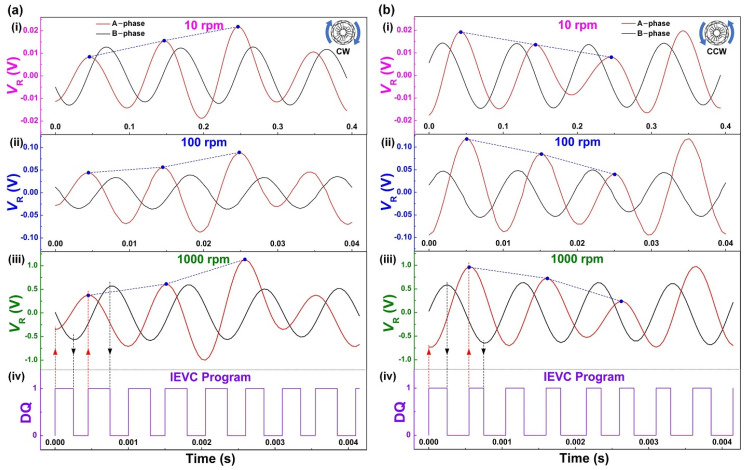
The electrical signals of the TRMS under different rotation speeds. (**a**) The CW rotation: 10 rpm, 100 rpm, 1000 rpm, and incremental extreme value counting (IEVC) program of 1000 rpm. (**b**) The CCW rotation: 10 rpm, 100 rpm, 1000 rpm, and IEVC program of 1000 rpm.

Figure 4a(i)–(iii) show the electrical signals in the CW rotation of the TRMS. In a cycle of electrical signals generated by three pairs of variable amplitude electrodes, the load voltage amplitude of A-phase increases sequentially at different speeds, thus the rotation direction is CW. Figure 4b(i)–(iii) show the electrical signals in the CCW rotation of the TRMS. Contrary to the output state in the CW rotation, when the load voltage amplitude of A-phase decreases sequentially at different speeds, the rotation direction can be judged as CCW. In addition, the electrical signals of the B-phase are the steady sinusoidal signal in both CW and CCW rotation, which has a phase difference of about π/2 from the electrical signals of the A-phase. The above experimental results are entirely consistent with the theoretical analysis results (Figure 2b,c). To reflect the rotation angle monitoring of the TRMS, an incremental extreme value counting (IEVC) program is performed by software. When the hybrid electrical signals reach the extreme value point (peak or valley) for the first time, the digital quantity (DQ) rises to “1”. Then, when it reaches the extreme value point (peak or valley) for the second time, the DQ drops to “0” and repeats the above steps to achieve incremental monitoring of rotation angle. It is worth noting that it is only related to the number of extreme value points in this counting program. The results analyzed by the IEVC program under the rotation speed of 1000 rpm are shown in Figure 4a(iv),b(iv). The above experiment results verify the feasibility that the direction and angle monitoring of the industrial-grade rotary motion can be realized by the amplitude characteristics of the hybrid electrical signals. Figure 4 shows that the output phase difference between the A-phase and B-phase has a small error. The reasons may be the assembly error of the prototype and the unstable delay caused by the equipment.

Moreover, the TRMS can monitor the rotation speed by the frequency of output signals. A periodic signal includes two pairs of adjacent interdigital electrodes, and the following equation is used to calculate the rotation speed *n*:

(1) $$n=\left(\frac{2\theta }{C_{0}}\right)ft_{0}$$

where *n* is the rotation speed of the rotor, *θ* is the interdigital electrode interval, *f* is the frequency of electrical signals, and *C*_0_ and *t*_0_ are constant numbers (*C*_0_ = 360° and *t*_0_ = 60 s). The interdigital electrode interval of the prototype *θ* is 3°. Therefore, the relationship between the rotation speed *n* and the frequency of electrical signals *f* of the prototype can be expressed as follows:

(2) n=f

The frequencies at different rotation speeds of 10–1000 rpm are acquired for linear fitting and their linearity and error rate are analyzed. The calculation equation of the error rate *δ* is as follows:

(3) $$\delta =\frac{\left|n-n_{\mathrm{Encoder}}\right|}{n_{\mathrm{Encoder}}}\times 100\%$$

where *δ* is the error rate of TRMS and *n*_Encoder_ is referenced rotation speed monitored by the encoder.

**Figure 5:**
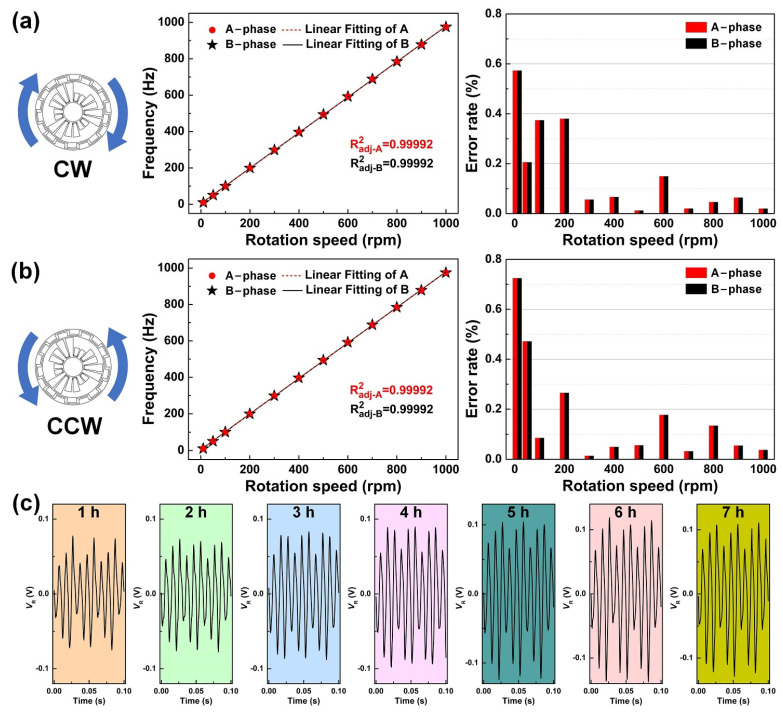
Rotation speed analysis and durability experiment of the TRMS. (**a**,**b**) The linearity and error rate of CW and CCW rotation. (**c**) Electrical signals acquired by the TRMS at different times.

The analysis results of the CW and CCW rotation are shown in Figure 5a,b, respectively. The TRMS shows good linearity between its rotation speed and frequency in the range of 10–1000 rpm, and the adjusted R square of the A-phase and B-phase are equal, at about 0.99992. The good linearity proves the excellent ability of the TRMS as a rotation speed monitoring sensor. Moreover, the rotation speeds calculated from the frequency of the TRMS are compared with the values measured by the encoder; the error rate can be controlled below 0.8%. Significantly, the TRMS shows better accuracy under high rotation speeds, and the error rate of the TRMS is less than 0.2%. The reason for this experimental phenomenon is that, when the rotation speed is higher, the load voltage of the electrical signal is larger, and the signal-to-noise ratio is better at this time.

Further, a durability experiment is performed on the TRMS. As shown in Figure 5c, after about 7 h of continuous testing, there is no significant electrical signal attenuation. The experiment process is equivalent to a continuous rotary motion of approximately 42,000 revolutions, proving that the TRMS is stable enough to perform well in long-term operation.

### 3.2. Demonstration and Application

**Figure 6:**
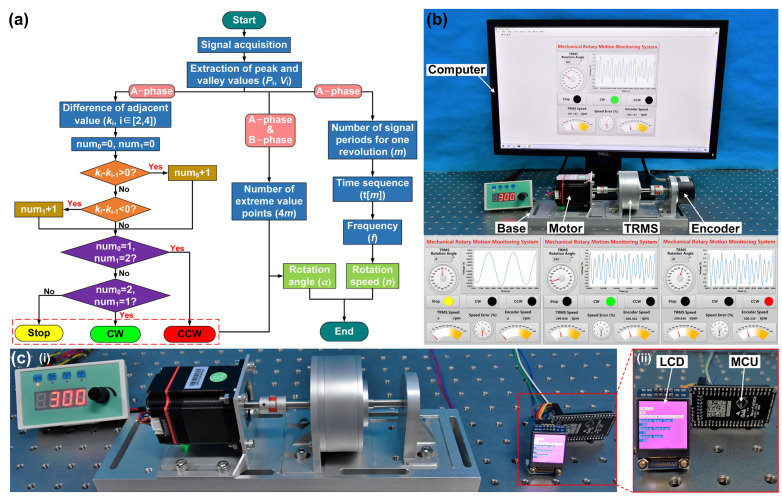
Application and demonstration of the TRMS in rotary motion monitoring. (**a**) Program flow chart of the signal process. (**b**) Mechanical rotary motion monitoring system and display function interfaces. (**c**) Industrial application of the IMS.

A mechanical rotary motion monitoring system program is developed by the LabVIEW software to realize the application of the TRMS. The flow chart of the signal processing program is shown in Figure 6a. Firstly, the difference between adjacent value *k*_i_ (i = 2~4) is calculated by the peak and valley values of the A-phase electrical signal, and then the three adjacent differences are compared to judge the rotation direction of the rotary motion. Secondly, the number of extreme value points (peak and valley) of the hybrid electrical signals is 4*m* to calculate the rotation angle (the value is four times the number of signal periods of A-phase for one revolution *m*). Finally, the rotation speed *n* is calculated by the number of signal periods of the A-phase for one revolution *m* and the frequency of the A-phase electrical signal *f*. The error rate at different rotation speeds can be displayed by comparing it with the value measured by the encoder in real time.

A rotation speed calibration experiment is performed on the TRMS, and the corresponding mechanical rotary motion monitoring system and functional display interface in the three different states are shown in Figure 6b. The demonstration of the system in Video S1 confirms that the TRMS can measure the rotary motion of the industrial-grade mechanical shaft system with high resolution. In addition, to realize the integration of the prototype, the TRMS is made into an IMS using modular electronic components. Figure 6c shows the industrial application of the IMS. The application process is given in Video S2. More importantly, the IMS verifies the feasibility of the TRMS for self-powered rotary motion monitoring and can meet the requirements of most industrial manufacturing applications.

## 4. Conclusions

In summary, we have proposed a TRMS with variable amplitude differential hybrid electrodes and designed an IMS based on the TRMS, digital signal processing method, and modular electronic components. The monitoring of industrial-grade rotary motion state is realized by the variable amplitude differential hybrid electrodes generating two-phase electrical signals simultaneously. The signal characteristics of the TRMS under different motion states are monitored in the experiment system. The results indicate that the TRMS can realize rotation speed measurement in the range of 10–1000 rpm and incremental rotation angle monitoring in the range of 360° with 1.5° of the resolution of the rotation angle. Moreover, the error rate of the rotation speeds calculated from the TRMS frequency can be controlled below 0.8%. Significantly, the error rate of the TRMS is less than 0.2% under the high rotation speeds. It has a high resolution and excellent linearity and can meet the requirements of most industrial manufacturing applications. Based on the above experience and the application, the TRMS can realize self-powered monitoring of industrial-grade rotary motion state. This work is of great significance to the development of triboelectric sensors in industrial automation sensing and monitoring.

## Data Availability

Not applicable.

## Supplementary Materials

The following are available online at https://www.mdpi.com/1424-8220/21/5/1713/s1, Video S1: The Demonstration of TRMS in Mechanical Rotary Motion Monitoring System; Video S2: The Industrial Application of TRMS in Integrated Monitoring System.
