# Supplementary material for: Triboelectric Rotary Motion Sensor for Industrial-Grade Speed and Angle Monitoring
Source: Sensors (Basel). 2021 Mar 2;21(5):1713. doi: 10.3390/s21051713 (PMC7958622; doi:10.3390/s21051713)
Supplement: Supplementary file 1 [file sensors-21-01713-s001.zip › Supplementary Files/Author Biographies.docx]

**Xiaosong Zhang** was born in Sichuan province, China, in 1996. He received the B.S. degree in mechanical engineering and automation from Changchun University of Technology, Changchun, China, in 2019，where he is currently working toward M.S. degree at the Department of Mechanical Engineering. His research interests include triboelectric nanogenerators, triboelectric sensors and piezoelectric actuators.

**Qi Gao** was born in 1995 Jilin province, majored in mechanical engineering and achieved the B.E. and M.S. degrees from Changchun University of Technology in 2017 and 2020. His research interests include triboelectric nanogenerators, triboelectric sensors and piezoelectric actuators.

**Qiang Gao** was born in Shanxi province, China, in 1994. He received the B.S. degree from Luoyang Institute of Science and Technology, Luoyang, China, in 2018. Now, He is studying for a master’s (M.S.) degree in mechanical engineering at Changchun University of Technology. His research interests include friction drive mechanism, piezoelectric energy harvesting, signal analysis and triboelectric nanogenerators.

**Dr. Xin Yu** is a visiting scholar in Beijing Institute of Nanoenergy and Nanosystems, Chinese Academy of Sciences, currently. He obtained the B.S. and Ph.D. degrees from College of Electronic Science and Engineering, Jilin University in 2009 and 2014, respectively. He was a visiting scholar in Department of Electrical and Computer Engineering, Michigan State University from 2017 to 2018. His interests are triboelectric nanogenerators, infrared gas sensing, and photoelectric detection.

**Prof. Tinghai Cheng** received the B.S., M.S. and Ph.D. degrees from Harbin Institute of Technology in 2006, 2008 and 2013, respectively. He was a visiting scholar in the School of Materials Science and Engineering at Georgia Institute of Technology from 2017 to 2018. Currently, he is a professor of Beijing Institute of Nanoenergy and Nanosystems, Chinese Academy of Sciences. His research interests are triboelectric nanogenerators, piezoelectric energy harvester, and piezoelectric actuators.

**Prof. Zhong Lin Wang** received his Ph.D. from Arizona State University in physics. He now is the Hightower Chair in Materials Science and Engineering, Regents’ Professor, Engineering Distinguished Professor and Director, Center for Nanostructure Characterization, at Georgia Tech. Dr. Wang has made original and innovative contributions to the synthesis, discovery, characterization and understanding of fundamental physical properties of oxide nanobelts and nanowires, as well as applications of nanowires in energy sciences, electronics, optoelectronics and biological science. His discovery and breakthroughs in developing nanogenerators established the principle and technological road map for harvesting mechanical energy from environment and biological systems for powering personal electronics. His research on self-powered nanosystems has inspired the worldwide effort in academia and industry for studying energy for micro-nano-systems, which is now a distinct disciplinary in energy research and future sensor networks. He coined and pioneered the field of piezotronics and piezophototronics by introducing piezoelectric potential gated charge transport process in fabricating new electronic and optoelectronic devices. Details can be found at: http://www.nanoscience.gatech.edu
