# Supplementary material for: Triboelectric Rotary Motion Sensor for Industrial-Grade Speed and Angle Monitoring
Source: Sensors (Basel). 2021 Mar 2;21(5):1713. doi: 10.3390/s21051713 (PMC7958622; doi:10.3390/s21051713)
Supplement: Supplementary file 1 [file sensors-21-01713-s001.zip › Supplementary Files/Highlights.doc]

**Highlights**

1. A novel triboelectric rotary motion sensor (TRMS) adopts hybrid electrodes with variable amplitude electrode and differential electrode.
2. The proposed TRMS can realize real-time monitoring of rotation speed, angle, and direction simultaneously. It has a high resolution and excellent linearity and can meet the requirements of most industrial manufacturing applications.
3. An integrated monitoring system based on the TRMS is applied to the measurement of industrial rotation motion, which further promotes the development of the TRMS in industrial application.
